# Autocatalytic Metallization of Fabrics Using Si Ink, for Biosensors, Batteries and Energy Harvesting

**DOI:** 10.1002/adfm.201804798

**Published:** 2018-11-09

**Authors:** Max Grell, Can Dincer, Thao Le, Alberto Lauri, Estefania Nunez Bajo, Michael Kasimatis, Giandrin Barandun, Stefan A. Maier, Anthony E. G. Cass, Firat Güder

**Affiliations:** ^1^ Department of Bioengineering Imperial College London London SW7 2AZ UK; ^2^ Laboratory for Sensors Department of Microsystems Engineering‐IMTEK University of Freiburg 79110 Freiburg Germany; ^3^ Department of Chemistry Imperial College London London SW7 2AZ UK; ^4^ Department of Physics Imperial College London London SW7 2AZ UK; ^5^ Chair in Hybrid Nanosystems Nanoinstitute Munich Faculty of Physics Ludwig‐Maximilians‐Universität München 80539 München Germany

**Keywords:** energy harvesting and storage, fabrics, paper, sensing, textiles

## Abstract

Commercially available metal inks are mainly designed for planar substrates (for example, polyethylene terephthalate foils or ceramics), and they contain hydrophobic polymer binders that fill the pores in fabrics when printed, thus resulting in hydrophobic electrodes. Here, a low‐cost binder‐free method for the metallization of woven and nonwoven fabrics is presented that preserves the 3D structure and hydrophilicity of the substrate. Metals such as Au, Ag, and Pt are grown autocatalytically, using metal salts, inside the fibrous network of fabrics at room temperature in a two‐step process, with a water‐based silicon particle ink acting as precursor. Using this method, (patterned) metallized fabrics are being enabled to be produced with low electrical resistance (less than 3.5 Ω sq^−1^). In addition to fabrics, the method is also compatible with other 3D hydrophilic substrates such as nitrocellulose membranes. The versatility of this method is demonstrated by producing coil antennas for wireless energy harvesting, Ag–Zn batteries for energy storage, electrochemical biosensors for the detection of DNA/proteins, and as a substrate for optical sensing by surface enhanced Raman spectroscopy. In the future, this method of metallization may pave the way for new classes of high‐performance devices using low‐cost fabrics.

## Graphical abstract

**Hydrophilic metallization on fabrics for new high‐performance devices** is demonstrated. A wide range of metals are deposited inside fabrics (paper, cotton) and nitrocellulose membranes, without modifying the surface. Coil antennas are then demonstrated for wireless energy harvesting, Ag–Zn batteries for energy storage, electrochemical biosensors for DNA/protein detection, and a substrate for surface enhanced Raman spectroscopy.

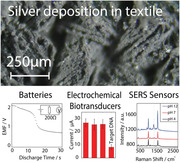

## 1 Introduction

Cellulose‐based woven and nonwoven fabrics are a mesh of overlapping fibers that are used in a wide array of applications, ranging from filtration to clothing.^1^, ^2^ Cellulose fabrics are porous, hydrophilic, chemically robust, and flexible materials with a high‐surface‐area and 3D geometry. Paper, a ubiquitous nonwoven fabric (costing ≈$0.001 dm^−2^), is commonly used for printed text and packaging. Unlike planar flexible substrates made of synthetic polymers (e.g., polyethylene terephthalate (PET)), high‐tech devices made of paper can contain electronic and microfluidic elements on the same (single) substrate.^3^, ^4^, ^5^ Although there has been substantial interest in using cellulose fabrics for emerging applications in electronics and sensing, deposition of electronic materials, especially metals, within the complex 3D geometry has been challenging without modifying the porous structure.^1^, ^6^, ^7^, ^8^, ^9^, ^10^, ^11^, ^12^

Incumbent techniques of printing metals on paper and other cellulose fabrics typically rely on micro/nanoparticle‐based inks that contain a polymer binder and organic solvents.^13^ These inks smoothen the rough surface and fill the pores, which prevent wicking by capillary action. Although the resulting electronic structures usually have sufficiently good electronic properties with high repeatability and conductivity, they are expensive (e.g., Ag ink typically over $10 per gram), brittle, and hydrophobic—a large drawback for (bio)chemical sensing and energy storage applications.^6^ Aqueous inks consisting of organic materials such as carbon nanotubes and poly(3,4‐ethylene dioxythiophene):poly(styrene sulfonate) have been applied as binder‐free printed electrical conductors for fabrics.^14^, ^15^, ^16^, ^17^ Although these inks preserve the 3D structure of the substrate, depending on the formulation, organic materials typically yield several orders of magnitude lower electrical conductance in comparison to metals and may not offer sufficient performance for applications such as interconnects and coil antennas. For use in batteries, low conductance would result in high internal resistance, and in the case of electrochemical sensing, it would decrease the sensor performance. Vacuum deposition methods can also be used to deposit organic and inorganic electrical conductors on fabrics.^18^ Physical vapor deposition methods such as thermal evaporation or magnetron sputtering can only produce conductive structures on the surface of the fabrics and they do not produce a conformal coating around the fibers within the fabric.^5^, ^19^, ^20^, ^21^ Furthermore, both physical and chemical vapor deposition methods involving vacuum processing (such as thermal evaporation or atomic layer deposition) are expensive and/or mostly limited to the deposition of thin films with sub‐micrometer thickness. The highly hygroscopic nature of cellulose fabrics also results in significantly longer pumping times to achieve high vacuum conditions needed for deposition or may cause unwanted parasitic reactions with the chemical precursors.^22^, ^23^

In contrast to the one‐step printing or deposition processes described above, fabrics can be internally metallized using two‐step or multistep electroless deposition strategies that generally do not necessitate polymer binders, although approaches are often tedious or require many steps, and are limited to specific metals.^24^, ^25^ Deposition inside fabrics results in high wettability metallized electrodes with a higher surface area (due to larger particle loading), which retains porosity. These characteristics enable rapid battery discharge and compact storage; the larger surface also results in a higher active electrode area for chemical sensing. Electroless deposition of copper on synthetic Teslin paper, for instance, has been demonstrated for inkjet‐printed electronic circuits.^26^ In this approach, paper was first coated with tin chloride (SnCl_2_). Next, a Ag precursor ink was inkjet printed on the substrate to act as a catalyst in an electroless deposition bath containing copper sulphate (CuSO_4_) and sodium hydroxide (NaOH), with a formaldehyde (CH_2_O) reducing agent. The Teslin paper is made of primarily polymer/silica, however, and multiple deposition steps are required, resulting in an expensive procedure less compatible with high‐volume manufacturing.

In this work, we introduce a two‐step process for the autocatalytic binder‐free metallization of fabrics, enabled by an aqueous Si‐based precursor ink. Using this method (Si ink‐enabled autocatalytic metallization (SIAM)), we have successfully grown Ag, Au, and Pt throughout the cross‐section or surface (depending on the application) of both woven and nonwoven cellulose fabrics without any (or minimal) alteration in the wetting behavior or porosity of the substrates. We used the SIAM method for fabrics for the following five applications: i) printing coil antennas on paper (for wireless energy harvesting using near‐field communication (NFC)), ii) fabrication of Ag–Zn batteries, iii) formation of electrochemical biosensors for the detection of nucleic acids, and iv) proteins. Rough metallic nanoparticle surfaces were also exploited as a v) plasmonic sensing substrate for surface enhanced Raman spectroscopy (SERS).

## 2 Results and Discussion

**Figure 1:**
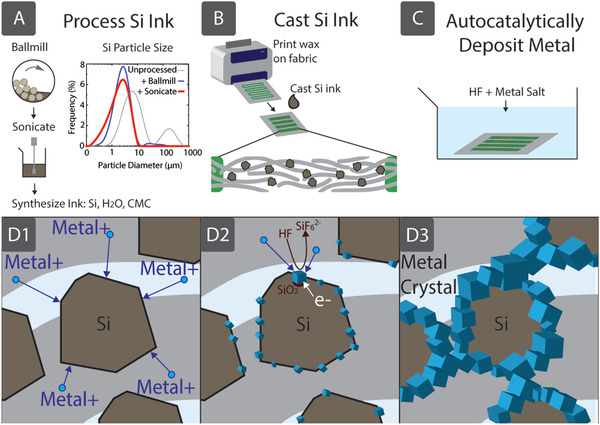
Metal fabrics are created by autocatalytic metallization of a Si precursor ink (SIAM). We processed Si micropowder into a precursor ink by ballmilling and ultrasonication until particle sizes match fabric pore sizes. To optimize for Whatman 4 paper, we used ballmilling to break large particles (from 1 mm diameter) and ultrasonication to reduce the median particle diameter to 2.5 µm, from 4.3 µm unprocessed. A) We then controlled viscosity by adding CMC. B) We inkjet printed wax barriers to confine the precursor ink to the required design on the fabric substrate. C) Next we placed the substrate in an autocatalyic bath containing HF and metal salts. D1) Metal^+^ ions are attracted to electrons in the Si valence band. D2) Electrons e^−^ in the Si are attracted to the deposited metal nuclei, catalyzing further reduction of metal^+^ ions. Si is subsequently oxidized near the metal nuclei, forming SiO_2_ that is etched away by the HF solution. D3) Metal^+^ ions in solution deposit preferentially on metal nuclei, which grow accordingly. This creates conductive percolation pathways throughout the entire fabric structure, formed around Si particles that sit within the fibres.

**Figure 2:**
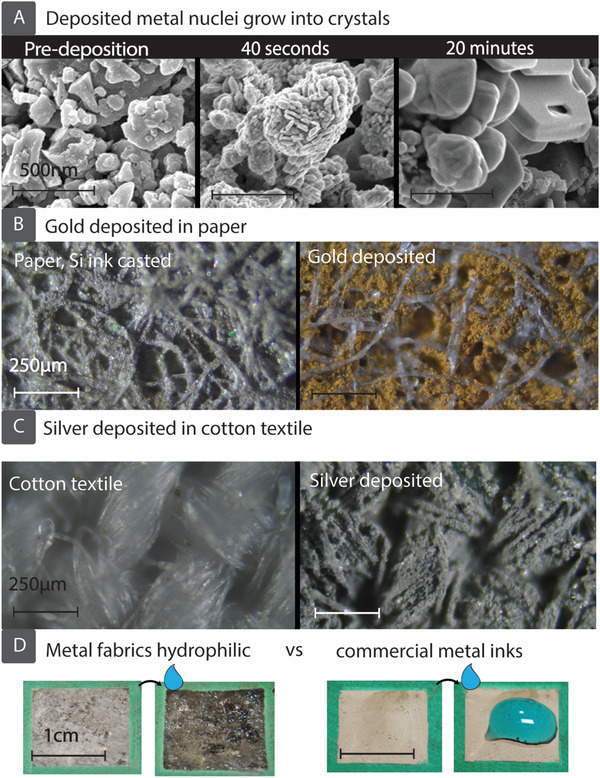
A) SEM images show Ag metallization during SIAM of paper. Si microparticles (predeposition) catalyze Ag nanoparticle deposition (visible after 40 s), which eventually grow in an Ostwald ripening process until Ag completely covers the Si particles (after 20 min), and conductive pathways are formed. Optical characterisation shows metals have been deposited throughout fabric microstructures, with B) Au in paper and C) Ag in cotton textile. D‐left) Metal fabrics are extremely hydrophilic and binder‐free, D‐right) whereas commmerically available metal nanoparticle inks are hydrophobic and require a binder.

Synthesis of the aqueous Si precursor ink, printing, and autocatalytic metallization of fabrics are illustrated in **Figure** **1**. Si micropowders, costing less than $0.001 per gram, were purchased from Magdev Ltd. (Swindon, UK) and processed using ballmilling and sonication to reduce particle size and create a more uniform distribution of particles (Figure 1A). After processing, particles with a mean diameter of 2.5 µm were dispersed in deionized water and mixed with carboxymethyl cellulose (CMC) on a magnetic stirrer for 3 h to create a Si:H_2_O:CMC precursor ink with ratio 2 g:20 mL:0.1 g. In this formulation, the CMC acts both as a stabilizer and viscosity modifier. Patterns of Si precursor ink are casted on hydrophilic cellulose fabrics by first printing a hydrophobic wax barrier, briefly annealing at 190 °C in air (causing it to wick through the porous cellulose structure) and then dispersing the aqueous Si precursor ink into the hydrophilic regions on the substrate (Figure 1B).^3^ Wax barriers enable confinement of the aqueous ink which presents a simple method to create patterns of Si precursor ink with commonly available, inexpensive equipment (in this work, a Xerox office printer). Some self‐aggregation of Si particles is observed, meaning that cellulose fibers are not uniformly covered (Figure S1, Supporting Information). After the evaporation of solvent (i.e., water) at room temperature the Si particles remain wrapped around the fibers and trapped within the fabric, which can be autocatalytically metallized (Figure 1C) by noble metals such as Ag, Au, Pt, etc. (see energy‐dispersive X‐ray (EDX) spectra in Figure S2, Supporting Information) by using a deposition bath containing a dilute aqueous solution of hydrofluoric acid (HF) and metal salts such as AgNO_3_, HAuCl_4_, and K_2_PtCl_6_. Once the Si ink is dry, rehydration does not remove the particles from the fabric, and the strength of the underlying fabric is only slightly reduced by HF exposure (Figure S3, Supporting Information). The process of autocatalytic metallization of Si particles and the formation of conductive networks of metals are illustrated in Figure 1D. Autocatalytic metal deposition is an electroless plating method that typically involves several chemical reactions in solution, without need for applying an external voltage. In this process, noble metal ions in close proximity to the Si particles attract electrons from the valence band of Si (Figure 1D_1_), forming nanoscale metal nuclei on the Si microparticles. The higher electronegativity of noble metals attracts electrons in the Si, causing the metal nuclei to become negatively charged. This catalyzes further reduction of metal ions (Figure 1D_2_) and the remaining metal ions in solution deposit preferentially on the existing metal nuclei, growing in an Ostwald ripening process (Figure 1D_3_). This process also increases oxidation of Si below the metal. The subsequent SiO_2_ is etched away by the HF, allowing the metal deposited to displace it.^27^, ^28^, ^29^, ^30^, ^31^, ^32^ This reaction is autocatalytic in that one of the reaction products, the noble metal itself, catalyzes further reductive deposition of metal ions.^33^ Scanning electron micrographs (SEM) recorded from a sample, predeposition (Si only), after 40 s and after 20 min autocatalytic metallization with Ag (**Figure** **2**A) also confirm that small Ag nuclei form on the Si crystals soon after immersion into the bath (40 s). After prolonged deposition, these nuclei grow into large crystals and then coalesce into even larger crystals (to reduce their surface energy) with a smooth surface.^34^, ^35^, ^36^ By inspecting the cross‐section of a Si‐wafer plated autocatalytically in a 1 m bath of AgNO_3_ (SEM image in Figure S4, Supporting Information), we estimate the Ag layer thickness increases at ≈2 µm min^−1^. Residual Si is present underneath the deposited metal, as confirmed by EDX measurement at high beam energy (20 keV), and thus high penetration depth (Figure S5, Supporting Information).

Figure 2B,C shows optical images of autocatalytically deposited Au and Ag in paper and cotton textile. The fabrics remain porous after the introduction of the Si ink and the subsequent metallization process. By modulating the wetting properties of the cellulose fabrics by wax printing, the SIAM process can also produce metallic structures both on the surface and through the cross‐section of fabric substrates, as shown in Figure S6 (Supporting Information). In contrast to commercially available conductive metal inks (such as Ag nanoparticle ink) that contain polymer binders, fabrics metallized by the process described here are extremely hydrophilic and drops of water placed on the sample are wicked spontaneously (see Figure 2D; Video V1 of the Supporting Information for comparison of wicking behavior).

**Figure 3:**
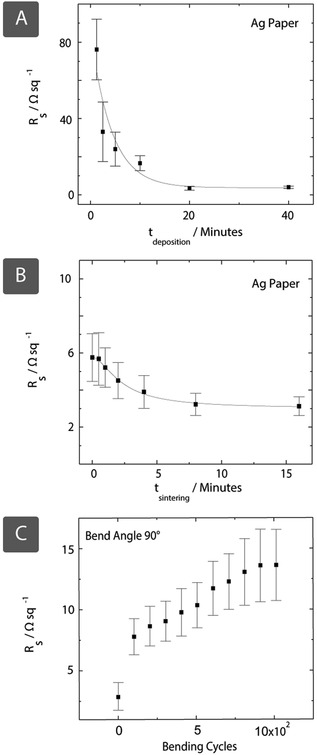
A) Sheet resistance *R*_s_ decreases with deposition time *t*_deposition_, as metal crystals grow larger and form more percolation pathways in an Ostwald ripening process. B) Rather than increasing deposition times, conductance may be increased more rapidly by sintering at 200 °C, with subsequent crystal growth causing metal particles to coalesce over time *t*_sintering_. We also report the durability of metallic paper (Ag) under cyclic strain (distance = 8 mm, angle = 90°) over 1000 cycles. C) The resistance increases significantly during the first 30 cycles. Number of samples *n* = 7 for deposition and sinter time, while *n* = 4 for bending cycles. Error bars correspond to the standard error.

We have measured the electrical conductance of Ag paper with varying durations of autocatalytic metallization (**Figure** **3**A). With increasing duration of metallization, the sheet resistance of the metallized substrate decreases reaching *R*_s_ = 3.5 ± 0.8 Ω sq^−1^ (*n* = 21) after 20 min. Further metallization does not appear to improve the conductance of the paper substrate. Increasing conductance with deposition time is most likely due to the formation of new conductive pathways within the fabric, because of the increasing size of metal crystals forming on the Si particles, as observed in Figure 2A. The conductance of the metallized fabric can alternatively be improved by sintering in a conventional oven at 200 °C. Sintering is a common postdeposition process for metals, where energy absorbed by the deposited metal particles causes them to coalesce, forming more conductive pathways.^37^, ^38^, ^39^ Rather than extending SIAM times, sintering in an oven can be deployed to decrease resistance toward the ≈3.5 Ω limit (as shown in Figure 3B) in less than 5 min. The nanoparticles create a thin metallic coating around the cellulose fibers, so fabrics retain their flexibility postdeposition (Figure 3C). In a 90° bending test, metallized paper was subjected to repeated bending up to 1000 times. In this experiment, the sheet resistance of the substrate increased from 7.5 to 12 Ω sq^−1^, probably due to the initial deformation of some of the loosely connected metallic connections within network of fibers. The increase in resistance seemed to have stabilized with increasing repetitions, which supports this assumption. With smaller bends, the metallized paper is better at retaining conductance, up to a bending distance of around 12 mm (Figure S7, Supporting Information). The ability of the metallized paper to retain conductance after bending indicates the potential of SIAM for applications requiring conformation to curved surfaces (for example, in wearables). In addition to fabrics, such as paper and cotton textiles, SIAM is also compatible with other 3D porous, hydrophilic substrates such as nitrocellulose membranes; the sheet resistances obtained for these materials are indicated in **Table** **1**.

**Table 1:** We have deposited Ag inside range of substrates via SIAM with varying sheet resistances. This process has been optimized for paper (*n* = 14 different samples) but cotton and nitrocellulose are also demonstrated (*n* = 7). Errors calculated using standard error

Substrate	Sheet resistance [Ω sq^−1^]
Paper	3.5 ± 0.8
Cotton	52 ± 15
Nitrocellulose	33 ± 15

### 2.1 Applications

#### 2.1.1 Energy Harvesting Using NFC

**Figure 4:**
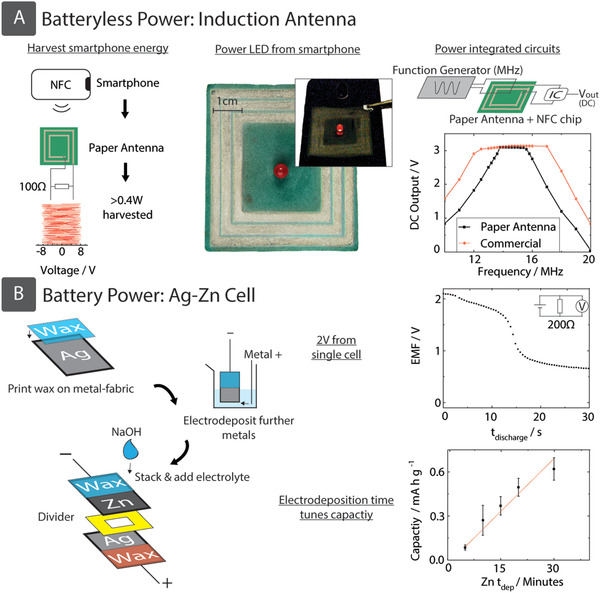
Fabric‐based electrochemical devices can be powered with batteries and/or embedded coil antennas fabricated by the SIAM method. A‐left) We have fabricated a Ag‐paper coil antenna, with metallization cost under $0.17, which was capable of harvesting 409.6 mW, based on a root‐mean‐squared voltage of 6.4 V harvested over a 100 Ω resistive load. A‐center) This was also sufficient to power a LED with a NFC enabled smartphone. The coil antenna was able to power an NFC integrated circuit to produce a regulated 3 V DC voltage (*V*_out_), intended for powering microcontrollers or sensors via NFC. A‐right) We compared this to a commerical etched antenna over a range of frequencies. Secondary metals may be deposited on top of SIAM fabrics by electroplating, enabling a variety of electrochemical cells such as Ag–Zn or Zn–Cu. B‐left) Wax printing provides a hydrophic barrier without hampering conductivity, enabling a contact point for the circuit. B‐right) We have fabricated Ag–Zn batteries capable of generating electromotive force (EMF) greater than 2 V from a single cell (shown here over 30 s discharge time *t*_discharge_), where controlling Zn electrodeposition time (Zn *t*_dep_) can tune the capacity. Error bars correspond to the standard error of measurements in 7 cells for each Zn deposition time.

NFC is an emerging radio frequency identification technology for short‐distance, secure, contactless transmission of data and power between two (one active and one passive) devices. NFC tags are generally fabricated through etching of a thin film of metal (mostly) vacuum deposited on a polymer substrate (typically PET), but the cost of a tag can be reduced substantially by using a paper substrate and additive methods such as SIAM for the deposition of antennas.^40^ We have applied the SIAM method for the fabrication of Ag coil (also known as loop or induction) antennas for NFC tags (**Figure** **4**A). We measured the power harvested by the coil antenna from an NFC‐enabled smartphone by connecting it to a purely resistive 100 Ω load and measuring the voltage induced with an oscilloscope across the load. The paper‐based Ag antenna was able to capture a root‐mean‐squared voltage of *V*_rms_ = 6.4 V from a smartphone, which corresponds to a harvested power of 409.6 mW. This was sufficient to power a light emitting diode (LED) and an NFC integrated circuit (IC) tag (NXP NTAG I^2^C plus), as illustrated in Figure 4A. The NXP NTAG I^2^C Plus chip can also be used to produce a regulated 3 V DC voltage (*V*_out_) from the RF signal transmitted from the smartphone that is intended for powering other devices, such as low‐power microcontrollers or sensors using NFC wirelessly. For comparison, we measured *V*_out_ when the NXP chip was connected to both a commercial printed‐circuit‐board and a paper‐based NFC antenna over a range of frequencies. Although the commercial antenna produced the maximum voltage over a wider range of frequencies, the paper‐based coil antenna with an unoptimized geometry and lower number of turns produced comparable results, hence it could power other devices through the use of an energy harvesting IC such as the NXP NTAG I^2^C.

#### 2.1.2 Energy Storage with Ag–Zn Battery

The SIAM method only allows autocatalytic deposition of noble metals like Ag, however, once a low‐resistance metallic fabric is created, it is possible to electroplate secondary, tertiary, etc. conductive materials (e.g., metals such as Zn, Ni, Cu) on the metallized substrate. We have electroplated Zn on Ag metallized paper by SIAM (Figure 4B) to form the anode of a flexible Ag–Zn battery with high pore density.^41^ The low resistance and high surface area of the metallized fabrics make them an ideal material for battery electrodes. It is, however, essential that a hydrophobic barrier (in this case, wax) is printed on the autocatalytically metallized substrate before electroplating to prevent the electrolyte from wicking through the paper to the source electrode (crocodile clip). Surprisingly we have discovered that, even though autocatalytically metallized paper printed with wax is impenetrable to aqueous solutions, crocodile clips could form firm low‐resistance ohmic contacts with the metallized substrate through the wax without any further procedures. We have vertically stacked 1.8 × 2 cm^2^ Ag metallized, bare (nonconductive separator) and Zn electroplated (30 min) paper (Figure 4B) to form the basic structure of the battery and added 250 µL of 2 m NaOH electrolyte through the top electrode to fully form a Ag–Zn battery (the electrolyte wicked spontaneously across the separator to the bottom electrode). Although there was high variation among the batteries produced (energy density values have standard deviation of 32%, after 30 min Zn electrodeposition), the best batteries had an open‐circuit potential greater than 2 V. Upon discharge over a 200 Ω load (discharge current of ≈10 mA) the voltage dropped from 2 to 1 V after 15 s, which corresponds to an energy density of 1.7 mA h g^−1^. With this power rating, the low‐cost Ag–Zn batteries produced could easily power a low‐power microcontroller with a low‐power sensing element and an liquid crystal display (LCD) screen for minutes to hours. The large variance in the quality of cells indicates considerable scope for optimization. The batteries produced are inherently lightweight and can be stored in dry form. Multiple cells can also be fabricated monolithically on a single sheet of paper, which can provide higher voltages and capacity.

#### 2.1.3 Electrochemical Biosensing of DNA

**Figure 5:**
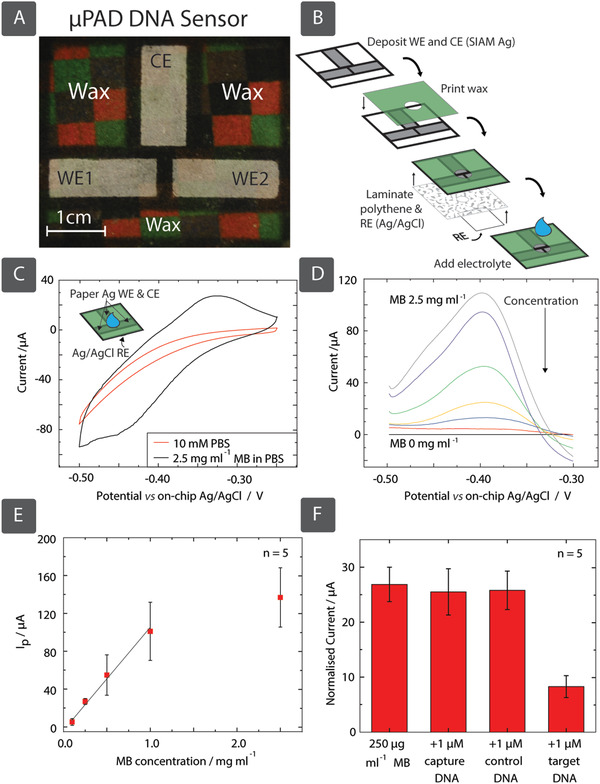
A) We have constructed a µPAD using SIAM to fabricate Ag paper counter and working electrodes. We printed wax on top with a circular hole in the center, and then laminated the bottom with polythene in a heatpress, also with a hole in the center. We fabricated the reference electrode by printing Ag/AgCl ink on the bottom of a second paper substrate. We then attached this to the polythene layer in the heatpress, with the top side of the RE paper substrate acting as a barrier between the RE and other electrodes. B) The result is a circular area in the µPAD's center capable of hydrophobically confining the electrolyte. C) We demonstrated the redox reaction of MB, D) calibrated for MB concentrations 0, 0.05, 0.1, 0.25, 0.5, 1, and 2.5 mg mL^−1^, and E) plotted against normalized current intensity *I*_p_. We have then shown normalized peak current intensities from SWVs recorded in MB solutions using the DNA biosensor in the presence of noncomplementary ss‐DNA (control) and complementary ss‐DNA (target). F) The current intensity is normalized with respect to the area. Error bars correspond to the standard error of measurements in 5 devices.

We have fabricated low‐cost electrochemical microfluidic paper‐based analytical devices (µPADs) with dual porous Ag working electrode (WE) and counter electrode (CE) formed with the SIAM process, as shown in **Figure** **5**A.^42^, ^43^, ^44^, ^45^ The Ag/AgCl pseudo‐reference electrode (RE) was printed on the rear side of a separate sheet of paper using a commercial ink and laminated immediately under the WEs (Figure 5B). We used a commercial RE ink because it did not penetrate the substrate (i.e., was deposited only on one side) and the hydrophilic front side of paper was used as a thin insulating separator between the WE and RE. SIAM produced porous electrodes are particularly good for electrochemical sensing for at least three reasons. The electrodes: i) have high electrical conductance comparable with thick‐film electrodes, ii) are hydrophilic, which may favor the immobilization of biomolecules, and iii) have a high electrochemically active surface area which is 2.1 times greater than the geometric area (Figure S8, Supporting Information), estimated using the Randles–Sevcik equation with experimentally measured gradient values of 0.67 ± 0.09. This in turn improves sensitivity. Dual WEs could also be used in combination to provide higher surface area for specific measurements, on‐demand. We used the fabricated devices for the detection of DNA biomarkers of *Mycobacterium avium* spp*. Paratuberculosis* (MAP). MAP is the causative agent of Johne's disease, a devastating disease with no cure affecting ruminant animals (such as cattle and sheep), and is also associated with Crohn's disease in humans.^46^, ^47^

The electrochemical approach (Figure S9, Supporting Information) we use for the detection of DNA involves the use of methylene blue (MB), a redox‐active reporter that binds specifically to the guanine bases during the hybridization of two single strands of DNA (ss‐DNA).^48^ Trapped molecules of MB between the target and capture DNA oligomers reduce the concentration of free MB in solution, leading to a decreased redox signal during electroanalysis. This approach can also be combined with polymerase chain reaction or isothermal amplification to enhance sensitivity.^48^, ^49^ We characterized the redox processes involving MB on the µPAD using cyclic voltammetry (CV) in concentrations ranging from 0 and 2.5 mg mL^−1^ (the potential was swept from −0.5 to −0.2 V at 100 mV s^−1^). The results indicate (Figure 5C) anodic and cathodic process at −0.33 and −0.46 V versus Ag/AgCl, respectively. We chose a potential window from −0.5 to −0.25 V versus Ag/AgCl, corresponding to the anodic process, to perform square wave voltammetry (SWV), which is a more sensitive method of analysis. Figure 5D illustrates recorded SWVs in MB solutions and phosphate‐buffered saline (PBS) with a range of concentrations from 0 to 2.5 mg mL^−1^. The calibration plot, peak current intensity versus MB concentration (Figure 5E), from SWVs from 5 different devices, shows a dynamic linear range from 50 µg mL^−1^ to 1 mg mL^−1^ (*R* ^2^ = 0.98). For the detection of MAP‐specific ss‐DNA, we measured the peak current intensities from SWVs recorded in 250 µg mL^−1^ MB solutions and 10 × 10^−3^ m PBS, before and after adding 1 × 10^−6^ m capture ss‐DNA, 1 × 10^−6^ m control ss‐DNA, and 1 × 10^−6^ m target ss‐DNA in this order. While the introduction of the capture and control oligomers did not produce a change in the electrochemical signal, addition of the target sequence resulted in DNA hybridization and subsequent trapping of the reporter, hence a reduction in the redox signal (Figure 5F). This experiment demonstrates the application of SIAM produced porous metal electrodes in selective, electrochemical quantification of DNA for the detection of pathogenic microbes.

#### 2.1.4 Electrochemical Biosensing of Proteins

**Figure 6:**
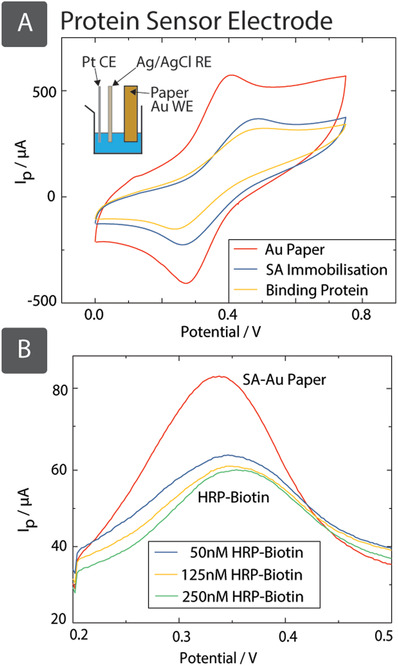
SIAM‐produced Au paper, coated with streptavidin (by dipping in 200 µL of 1 × 10^−6^ m SA, in 5 × 10^−3^ m Borax buffer for 2 h), has successfully immobilized 50 × 10^−9^ m of biotinylated protein (HRP). A) CVs of Au paper electrodes in 2 × 10^−3^ m FCA in PBS, with scan rate 100 mV s^−1^, demonstrate a decrease in current upon coating with SA, and a further decrease upon binding with biotinylated‐HRP. After SA was immobilized on the surface, oxidation and reduction peaks were shifted from 0.438 to 0.532 V and from 0.288 to 0.219 V, respectively. Binding of the biotinylated‐HRP to the immobilized SA caused a further small drift in peak separation to 0.57 and 0.20 V. B) SWV scans with increasing concentrations of biotinylated‐HRP are shown. A concentration of 50 × 10^−9^ m decreases the peak current intensity *I*_p_ by 45%, but higher concentrations did not lead to further drop in signal.

Au is commonly used for noncovalent immobilization of biomolecules, such as antibodies or other proteins on electrodes. We have therefore produced SIAM‐based Au paper WEs for electrochemical biosensing of proteins, in this case the enzyme horseradish peroxidase (HRP), based on the affinity reaction of streptavidin (SA) with biotin (Figure S10, Supporting Information). The exceptionally strong affinity and specificity of the biotin and SA have long been exploited for protein and nucleic acid labeling and immobilization, forming the basis of a large range of robust and sensitive biosensors. SA can be immobilized on a WE by adsorption, providing higher surface density than covalent attachment.^50^ We immobilized SA (i.e., recognition element) on the Au metallized paper (WE) by physical adsorption in a three‐electrode electrochemical cell (**Figure** **6**A, inset) with a Pt wire and Ag/AgCl electrode as the CE and RE, respectively. The effectiveness of the immobilization of SA and its interaction with biotin‐HRP can be tested by the use of an electroactive reporter, such as ferrocene carboxylic acid (FCA). When the number of proteins on the surface of the electrode increase due to immobilization or binding, the rate of diffusion of the reporter to the electrode also decreases, resulting in a decrease in the electrochemical signal.^51^

Cyclic voltammograms in Figure 6A reveal a marked decrease in the peak current intensity of 40% (both anodic and cathodic processes) when the electrode was coated with 1 × 10^−6^ m SA in 5 × 10^−3^ m Borax buffer for 2 h. This effect is observed by sweeping the potential at any scan rate (Figure S11, top and middle, Supporting Information). Once biotinylated‐HRP is added to the SA‐coated electrode the intensity decreases another 30%. This indicates that SA can be effectively immobilized on the surface of the electrode and capture the target complex. The amount of biotinylated protein required to cover all the available binding sites was studied by SWV with increasing volumes of 5 × 10^−6^ m biotin‐HRP from 2 to 10 µL. A volume of 2 µL, which corresponds to a concentration of 50 × 10^−9^ m of biotin‐HRP, is enough to decrease the peak current intensity by 45% (Figure 6B) instantly. The electrochemical signal, upon addition of the target complex, remains stable even after 10 min (Figure S11, bottom, Supporting Information) and the current intensity does not decrease for higher concentrations of biotin‐HRP complex. This on–off proof‐of‐principle sensor demonstrates the usefulness of SIAM produced metallic fabrics for the immobilization and detection of proteins, and that they can be used high‐performance sensing electrodes in electrochemical biosensors.

#### 2.1.5 Optical Chemical Sensing with SERS

Finally, the metallic fabrics produced by SIAM were used as a high‐surface area SERS substrate for optical, noncontact measurement of pH. SERS‐active plasmonic substrates increase characteristic Raman spectra by 10^8^ but are typically prepared by expensive physical deposition methods to create Ag, Au, Cu surfaces, on planar substrates with a nanoscale topography.^52^ SIAM‐based hydrophilic metallic fabrics can be used as a low‐cost, ultrahigh surface‐area alternative to conventional SERS substrates.^53^

**Figure 7:**
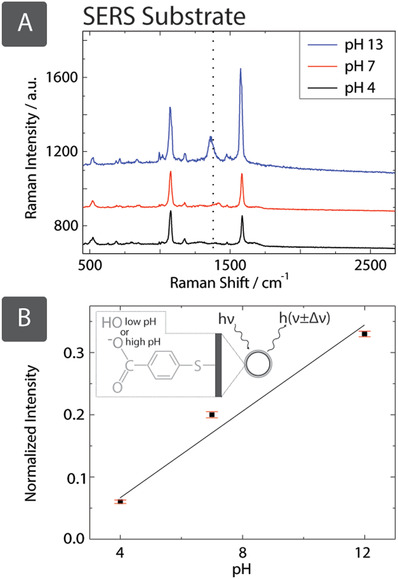
A) We have demonstrated that Au paper substrates produced by SIAM are an effective low‐cost substrate for SERS. After being functionalized with 4‐MBA (modified with a thiol ligand by submersing in a 0.1 m NaOH solution with 1 × 10^−3^ m 4‐MBA for 24 h), the characteristic peaks typical of its aromatic core are observed at 1100 and 1590 cm^−1^ in the Raman signal, using a 1.2 mW laser at 633 nm. This has been applied to pH measurements, with deprotonation of the carboxylic acid end group of 4‐MBA creating the characteristic peak at 1430 cm^−1^ for increasing pH. B) Solutions of varying pH were prepared using HCl and NaOH, in which the sample was submerged for deprotonation. Intensities of the pH‐dependent peak at 1430 cm^−1^ have been normalized with respect to the characteristic 4‐MBA peak at 1590 cm^−1^, showing a linear correlation between intensity and pH. Error bars are the standard error of measurements from 6 locations on the same sample.

We produced paper‐based Au and Ag plasmonic substrates for SERS and functionalized with 4‐Mercaptobenzoic (4‐MBA). The presence of 4‐MBA was confirmed by its characteristic peaks at 1100 and 1590 cm^−1^ in the Raman spectra (Figure S12, Supporting Information). Next, we used the Au paper for the noncontact measurement of pH, which is important in biological applications.^54^ Deprotonation of 4‐MBA due to changes in pH gives rise to the vibrational mode at 1430 cm^−1^ (**Figure** **7**), the amplitude of which can be used to estimate pH.^55^ This peak increased linearly (*R* ^2^ = 0.98) with the increasing pH between 4 and 12 confirming that metallic substrates produced by SIAM can be used for noncontact sensing of pH.

## 3 Conclusion

In conclusion, we have presented an electroless autocatalytic deposition technique capable of depositing a wide range of metals inside cellulose fabrics such as paper, cotton textiles, and even other 3D hydrophilic materials such as nitrocellulose membranes, using a precursor silicon ink. The process is sufficiently simple and can be performed in most academic and industrial facilities. The metallic fabrics produced are highly conductive, electrocatalytic, hydrophilic, flexible, porous, and ultralow cost (see the Supporting Information for cost comparison between SIAM and commercial Ag particle ink). We used the metallic fabrics produced by SIAM in wireless power transmission, energy storage, electrochemical sensing of DNA/proteins, and optical, noncontact plasmonic sensing with SERS.

We have discovered that deposition times below 20 min are optimal, although this can be lowered by sintering, and future work could explore deposition at higher temperatures. While the average sheet resistance of Ag deposited on paper was 3.5 ± 0.8 Ω sq^−1^, multiple deposition runs could decrease this and some of our preliminary tests yielded resistance values under 1 Ω sq^−1^. There is clear scope for an optimized SIAM process enabling fabric electronics with metallic interconnects and vias, integrated with microfluidics. An inherent disadvantage of the SIAM technique is the dependence on HF, and the consequential safety precautions that must be taken. Data presented here have been obtained using 5% HF, although the SIAM method has also been successfully performed with concentrations as low as 0.5% HF, in line with concentrations found in household cleaning products.^56^ Future work will determine optimal deposition parameters for such low HF concentrations. The potential of SIAM for deposition of multimaterial composites, using sequential deposition or mixtures of metal salts in HF, is another avenue for exploration.

The SIAM process has the potential to replace the etching methods used in the fabrication of coil antennas. When optimized, SIAM produced coil antennas that could cost as little as $0.001, which currently cost up to $0.05 using chemical etching processes. Metallic fabrics produced by SIAM are flexible and may allow fabrication of monolithically integrated battery‐powered wearable textile devices that can continuously perform sensing. Such devices could be charged wirelessly and may contain complementary metal‐oxide‐semiconductor (CMOS) electronics for data processing and wireless data transmission through NFC to a smartphone (charging and communication via the coil antenna).

Integration of a low‐cost power source (i.e., printed batteries) with electronics and microfluidic electrochemical or optical sensing elements—produced by SIAM—would also enable construction of fully integrated use‐and‐dispose devices. These devices could detect pathogens, diseases, explosives, or measure quality of water, food, soil, etc. at the point‐of‐need. Such instruments would be particularly useful in low‐resource settings in which a single point measurement is generally sufficient. Although, in this report, we have demonstrated the versatility of SIAM (and the metallic fabrics produced) in a few applications, we believe this is just a small sample and the potential applications for the method described are many.

## 4 Experimental Section

*Materials*: Paper substrate—Whatman 4 Qualitative Filter Paper 1004–185—was purchased from General Electric. Silver nitrate and hydrofluoric acid (50%) were obtained from Sigma‐Aldrich, UK. Silicon metal powder was from Pilamec UK Ltd and Zn metal sheet from Sanying Ltd.

*Silicon Precursor Ink*: Silicon powder was mixed with deionized water (0.1 g mL^−1^) and ground in a Capco 12VS rolling ballmill for 48 h. Ink was then ultrasonicated in a Branson Digital Sonifier 450 for 40 min, with an output power of 240 W. CMC was added with a ratio 1 g CMC per 100 mL Si ink.

*Autocatalytic Deposition (SIAM) Bath*: Fabric substrates with printed patterns of Si were submerged in the autocatalytic bath, which consisted of hydrofluoric acid (50 mL of 5%) mixed with silver nitrate (2.5 mL of 1 m), for 20 min. Gold was autocatalytically deposited by substituting silver nitrate for gold chloride trihydrate, and platinum using potassium hexachloroplatinate(IV) at the same concentrations and deposition times. Ag deposition on paper was performed, under the same conditions except with 0.5% HF concentration, yielding sheet resistance, *R*_s_ = 88 ± 30 Ω sq ^−1^. All SIAM was performed for 20 min unless otherwise stated.

*Paper Devices*: Wax designs were printed with a Xerox ColorQube 8580 wax printer on Office Depot transparent sheets and transferred to paper substrates with a Vevor HP230B heat press at 180 °C. Silicon precursor inks were pipetted and then allowed to dry at room temperature. Devices were washed with deionized water, submerged in the SIAM bath, washed again, and then allowed to dry at room temperature.

*Electrical and Material Characterization*: Particle size distribution measurements were made with a Malvern Masterizer 2000 laser diffraction particle size analyzer, with 50 mg Si particles dispersed in deionized water (20 mL), measured at pump speed of 1750 rpm.

Optical microscope images have been taken on a Brunel SP202XM metallurgical microscope connected to a Nikon D3200 camera. SEM images were acquired using a Sigma 300 and EDX measurements, a LEO Gemini 1525 electron microscope at 5 keV electron beam energy unless otherwise specified.

Conductivity measurements were made using rectangle samples, 0.5 × 4 cm^2^, connected with crocodile clips on flat aluminum foil contacts, outputting to a Tenma T2‐7730A multimeter. Seven samples have been made for each measurement, with results averaged and standard error used. For sintering measurements, seven samples were placed in a Genlab OV General Purpose Oven at 200 °C, with measurements made at time intervals shown in the figure using a Tenma T2‐7730A multimeter. Contacts were made using crocodile clips and foil, to minimize abrasion of the sample.

*Bending Tests*: Tests were performed on an Instron 3360 Series using Bluehill 3 software and a 1 kN load cell, bending angles were calculated using a camera and imageJ software. Samples were placed under cyclic strain (bending distance = 8 mm, angle = 90°) for 1000 cycles.

*Fabrication and Characterization of NFC Coils Antennas*: Coil antennas were designed with wax barriers as described above, prior to SIAM. We connected the antenna across a 100 Ω resistor and placed it on the back of an NFC‐enabled phone. The voltage induced across the resistor was measured with a Tektronix TBS 1052B‐EDU digital oscilloscope. An LED was attached to the antenna using Ag conductive epoxy from MG Chemicals, with photos taken on a Nikon D3200 camera. A driver signal was swept from 10–20 MHz using a Feeltech Dual‐channel Function Generator, and the induction through our SIAM paper antenna was compared to that of a commercial NXP NTAG I^2^C plus PCB antenna board.

*Electrodeposition*: Zn was electrodeposited in NH_4_Cl (100 mL, 1 m) electrolyte upon Ag paper metallized by SIAM. The positive electrode was Zn metal sheet, and the negative electrode was SIAM‐produced Ag paper. Further wax barriers were printed on top of the metallized paper to prevent the electrolyte from contacting the source electrode. Electrodeposition was performed at a constant voltage of 1 V. The SIAM electrode was then washed with deionized water and dried gently with paper towel, before immediate use in battery.

*Battery Fabrication and Characterization*: Ag and Zn electrodes were separated by a bare paper barrier. Batteries were laminated with polyethylene in the heat press. A hole was pierced in the polyethylene prior to lamination, through which the electrolyte (250 µL of 2 m NaOH) was added. The circuit was completed over a 200 Ω resistive load, with voltage measured using a Tenma T2‐7730A multimeter. The energy density was calculated by integrating the first 15 s of the discharge curve, where the current was above 5 mA.

*DNA Sensing µPAD*: For the evaluation of the sensing device, cyclic and square wave voltammetry analyses were performed at room temperature with a handheld potentiostat PalmSens3 (PalmSens BV, The Netherlands) with the supplied PSTrace 5.3 software in a three‐electrode setup. Prior to the DNA measurements with SWV, the electrode surfaces were pretreated with cyclic voltammetry to ensure clean surface. The pulse amplitude used for SWV analysis was 50 mV with a step potential of 2 mV a frequency of 10 Hz, and a potential range between −0.2 and −0.5 V. All chemicals used in this work were purchased from Sigma‐Aldrich, UK. The oligonucleotides, including a capture DNA (cDNA) 5′‐ttg gcc gat gga ggc gag gt‐3′, a target DNA (tDNA) complementary to cDNA 5′‐acc tcg cct cca tcg gcc aa‐3′, and a control DNA (contDNA) 5′‐aac cca tgg aat tca gtt cg‐3′, were purchased from Biomers.net GmbH, Germany. All solutions and dilutions were prepared in PBS (10 × 10^−3^ m) solution at pH 7.4, containing sodium chloride (138 × 10^−3^ m), and potassium chloride (2.7 × 10^−3^ m).

*Electrocatalytically Active Area*: Calculated using the Ranles–Sevcik equation. See the Supporting Information for details.

*Transducer for Biosensing*: SA, HRP, FCA, Avidin, 4′‐hydroxyazobenzene‐2‐carboxylic acid (HABA) were from Sigma‐Aldrich. EZ‐Link N‐hydroxysuccinimidobiotin and Zebra spin desalting columns (7K molecular weight cut‐off (MWCO)) were from Thermo Fisher Scientific. Amicon Ultracel‐10 was from Millipore. Other common chemicals were obtained from Sigma‐Aldrich unless specified. The reactive NHS‐biotin was conjugated to lysine residues of HRP in PBS (10 × 10^−3^ m). A series of different NHS‐biotin:protein molar ratios were performed to achieve the desired biotinylated level (close to 1 biotin per enzyme molecule). The excessive NHS‐biotin was removed using the Zebra spin column. The biotinylation was quantitated using HABA pulling assay. The biotin‐conjugated HRP was then transferred to the electrochemical reaction solution (ECRS) containing FCA (2 × 10^−3^ m) in PBS using the Amicon column. Concentrations of biotinylated‐HRP were determined using absorbance at 402 nm with an extinction coefficient value ε_402_ = 102 × 10^3^ m ^−1^ cm^−1^.^57^ A strip of the AuPE (≈3 mm wide and 15 mm long) was dipped into a well of a 96‐well microtiter plate containing 200 µL of 1 × 10^−6^ m of SA in 5 × 10^−3^ m Borax buffer for 2 h. The AuPE strip was then washed by consecutive dipping for 2 min each in 3 wells of ECRS (300 µL).

*SERS Preparation and Measurement*: Surface of metal nanoparticles inside paper was modified with a thiol ligand by submersing in a NaOH solution (0.1 m) with 4‐MBA (1 × 10^−3^ m) for 24 h to functionalize surface prior to Raman measurements were then made in a confocal Raman microscope (WiTEC) with a bright field objective (Zeiss 100× NA 0.9), with 1.2 mW laser at 633 nm. Solutions of pH 3, 7, and 13 were prepared using HCl and NaOH, in which the sample was submerged for 2 h to protonate the carboxyl moiety of MBA.

## Conflict of Interest

The authors declare no conflict of interest.

## Supporting information

Supplementary

Supplementary
