# Supplementary material for: Autocatalytic Metallization of Fabrics Using Si Ink, for Biosensors, Batteries and Energy Harvesting
Source: Adv Funct Mater. 2018 Nov 9;29(1):1804798. doi: 10.1002/adfm.201804798 (PMC7384005; doi:10.1002/adfm.201804798)
Supplement: Supplementary file 1 — Supplementary [file ADFM-29-1804798-s002.pdf]

Copyright WILEY-VCH Verlag GmbH & Co. KGaA, 69469 Weinheim, Germany, 2018.

ADVANCED FUNCTIONAL MATERIALS

Supporting Information

for *Adv. Funct. Mater.,* DOI: 10.1002/adfm.201804798

Autocatalytic Metallization of Fabrics Using Si Ink, for Biosensors, Batteries and Energy Harvesting

Max Grell, Can Dincer, Thao Le, Alberto Lauri, Estefania Nunez Bajo, Michael Kasimatis, Giandrin Barandun, Stefan A. Maier, Anthony E. G. Cass, and Firat Güder**

Supporting Information

Autocatalytic Deposition of Metals in Fabrics using Si Ink

Max Grell[1*], Can Dincer[1,2], Thao Le[3], Alberto Lauri[4], Estefania Nunez Bajo[1], Michael Kasimatis[1], Giandrin Barandun[1], Stefan A. Maier[4,5], Tony Cass[3], Firat Güder[1*]

1. Department of Bioengineering, Imperial College London, London, UK SW7 2AZ

2. Department of Microsystems Engineering – IMTEK, University of Freiburg, 79110, Freiburg, Germany

3. Department of Chemistry, Imperial College London, London, UK SW7 2AZ

4. Department of Physics, Imperial College London, London, UK SW7 2AZ

5. Chair in Hybrid Nanosystems, Nanoinstitute Munich, Faculty of Physics, Ludwig-Maximilians-Universität München, München, Germany

*Corresponding Authors:

m.grell15@imperial.ac.uk

guder@imperial.ac.uk

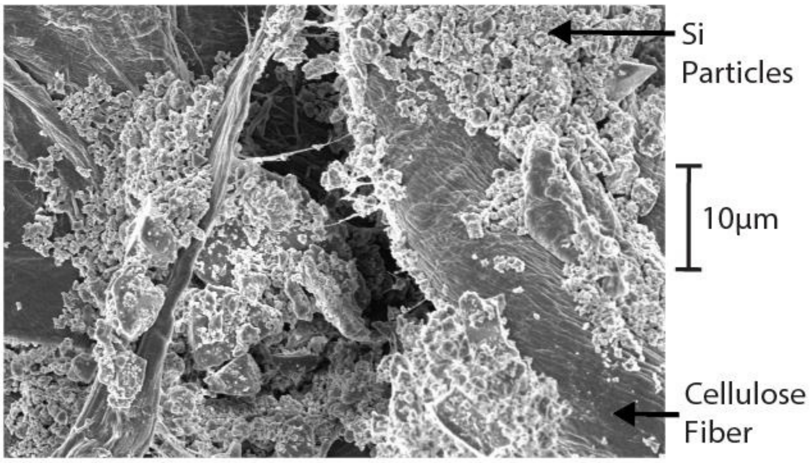

Figure S1: SEM image of Si ink drop-casted onto cellulose paper. Self-aggregation of Si particles is apparent amidst the partial coverage of cellulose fibers, which is inhomogeneous at micron scale.

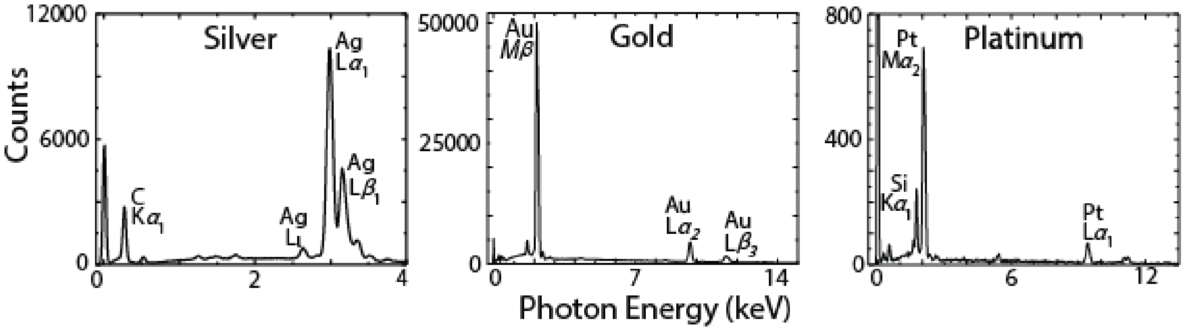

Figure S2: Energy-dispersive X-ray spectroscopy spectra of Ag, Au and Pt deposited with the SIAM method inside paper, taken with a SEM at 5keV beam energy.

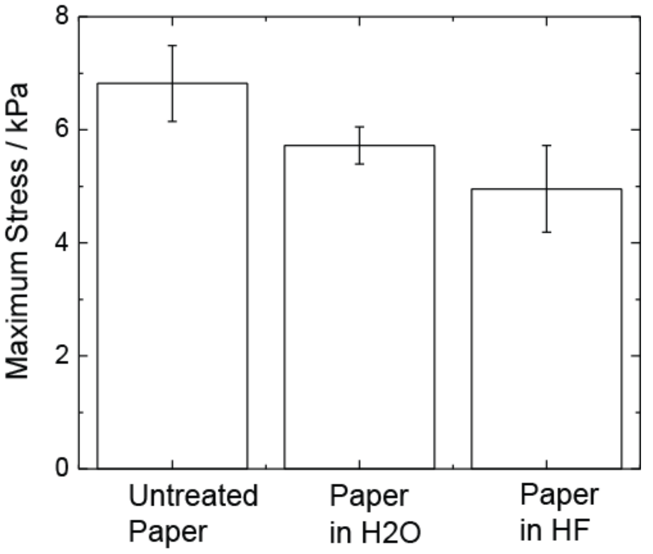

Figure S3: The maximum tensile stress withstood by paper before breaking decreases after 20 min. soaking in water, and after soaking in HF (5%). The maximum strain before breaking observed for each sample was similar (0.05±0.01). Samples measured 30×5 mm^2 and 5 repeats were done for each case, with errors corresponding to the standard deviation. Mechanical tests were performed using an Instron 5543 tensile tester (1 kN load cell, Bluehill 3 software).

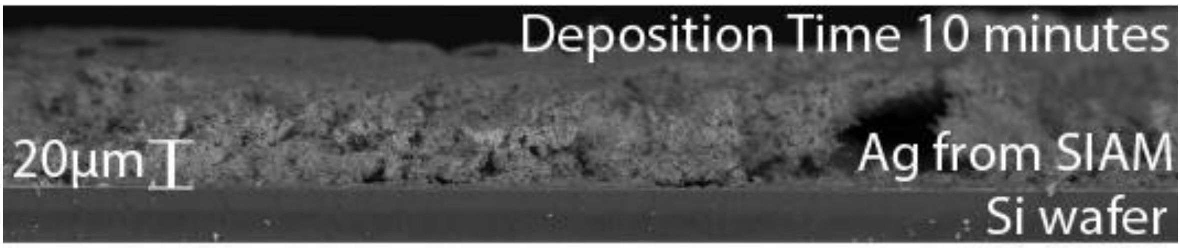

Figure S4: SEM image of the cross-section of a Si wafer (purchased from Siegert Wafer), with Ag deposited on the surface autocatalytically, in a bath of $AgNO_3$ (1M) and HF (5%). The Ag layer is approximately 20 µm thickness and therefore we approximate a deposition rate of 2 µm min^{-1}.

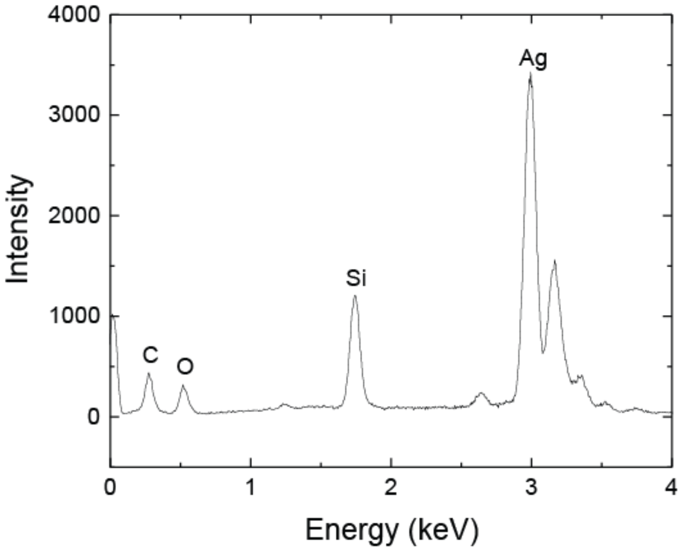

Figure S5: Energy-dispersive X-ray spectroscopy spectrum of Ag deposited by SIAM on paper, taken in a SEM at 20 keV beam energy, demonstrate the presence of residual Si.

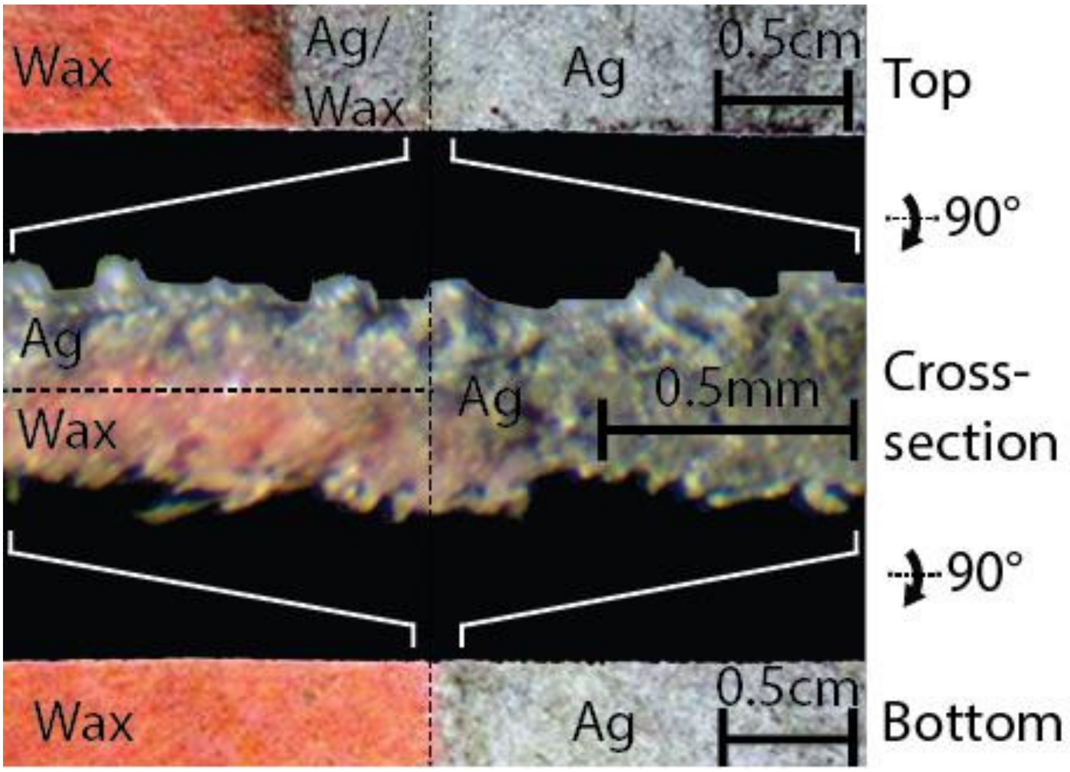

Figure S6: In the bottom photograph the left half of a strip of paper is printed with orange wax and the right half with Si ink. Ag is then electrolessly deposited on the Si ink (by SIAM method). On the top photograph, Ag is also deposited over the wax in the middle section. The

optical microscope image of the cross-section shows Ag is deposited above the wax on the left side, whereas on the right side it is deposited through the full cross-section of the paper.

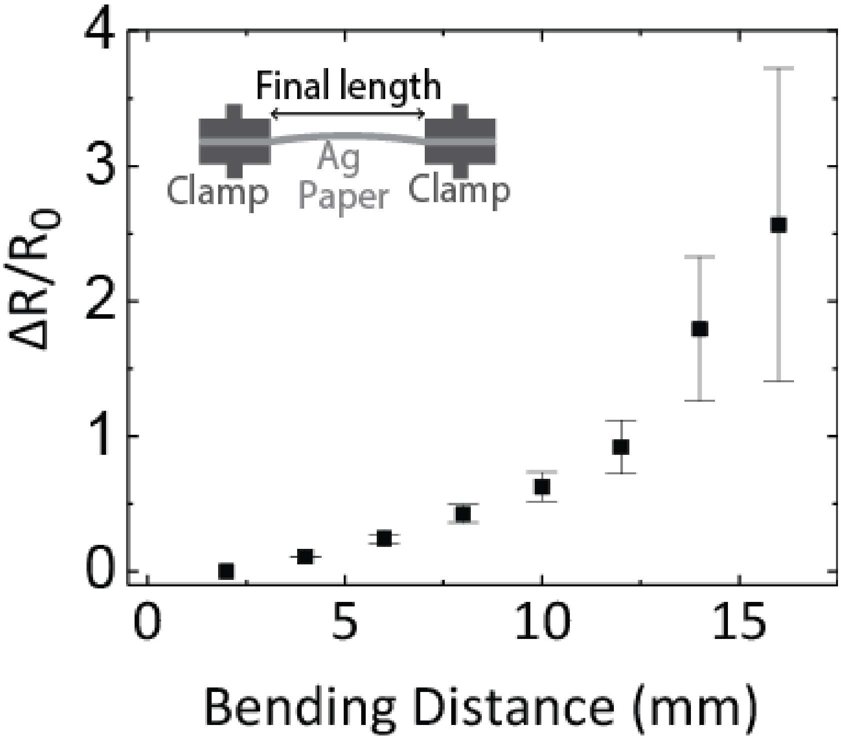

Figure S7: Metallized paper substrates have been bent by attaching between two clamps, which were then drawn closer together (S3 inset diagram). The length was measured before (initial length) and after bending (final length). Bending distance is the difference between these lengths. The corresponding change in resistance ΔR, normalized to the sample resistance before bending R_0, shows a gradual linear increase approaching 12 mm bending distance, at which point the conductive pathways began to separate, and higher variance is obtained. There were n = 6 repeats for samples from 2 to 14 mm bending distance, and n = 2 repeats for 16 mm bending distance as the samples began to break.

Calculation of Electrocatalytically Active Area: The dependence of the peak current on the scan rate was evaluated by cyclic voltammetry sweeping the potential from –0.8 to +1.0 V vs Ag/AgCl at 10, 25, 50, 75, 100, 150 and 200 mV s^{-1}. As the inset in **Figure S4** shows, a standard Ag/AgCl reference electrode (Fisherbrand Accumet model from FisherScientific, NL) and a graphite rod (Sigma Aldrich), as counter electrode, were placed close to the surface of the Ag-Si paper electrode (2 cm^2 of geometric area) which acts as working electrode. The electrochemical cell was connected to the potentiostat (PalmSens3 model from PalmSens, UK) with crocodile clamps. Then, 1 mL of 1 mM $K_4Fe(CN)_6$ solution in 0.1 M KCl was slowly added on the paper electrode, wetting the whole electrochemical cell and avoiding solvent evaporation during the experiment. According to the Randles-Sevcick equation for a flat electrode and for diffusion-controlled processes at 25 °C.[1-4]

$$i_p = (2.69\times10^5)\, n^{3/2} A D^{1/2}\, C v^{1/2}$$

Where i_p is the peak current (A), n is the number of electrons transferred (n = 1 for ferrocyanide), A the effective area of the electrode (cm^2), D is the diffusion coefficient of ferrocyanide in aqueous solutions (6.50×10^{-6} cm^2 s^{-1}), C is the concentration (1×10^{-6} mol cm^{-3}) and v is the scan rate (V s^{-1}).

Cyclic voltammograms, as those shown in Figure S4, were recorded using five different paper substrates without washings between scans. The gradient of the logarithmic plot peak current intensity vs the scan rate was 0.67±0.08 (R^2 = 0.997) and 0.67±0.09 (R^2 = 0.996), for anodic and cathodic processes, respectively. That values did not correspond to the value of 1 as expected for adsorbed electroactive species, or to the value of 0.5 associated with the semi-infinite diffusion of the electroactive species to the electrode. Compton et al. observed a similar behavior for the detection of nicotine on pyrolytic graphite electrodes modified with layers of multiwalled carbon nanotubes.[5] They obtained a gradient of 0.69 which is

indicative of a possible mixed mass transport regime with thin-layer diffusion within the porous conductive layer, and semi-infinite diffusion outside the layer in solution. Ag-Si paper electrode is also a porous material with channels and pores of variable dimensions and mixed behavior can be observed. Readjusting the Randles-Sevcik equation with the experimental gradient values, calculated effective areas were 8.2±0.4 cm^2 and 8.6±0.6 cm^2 from anodic and cathodic data, respectively. These values are at least the double of the geometric area (4.0 cm^2).

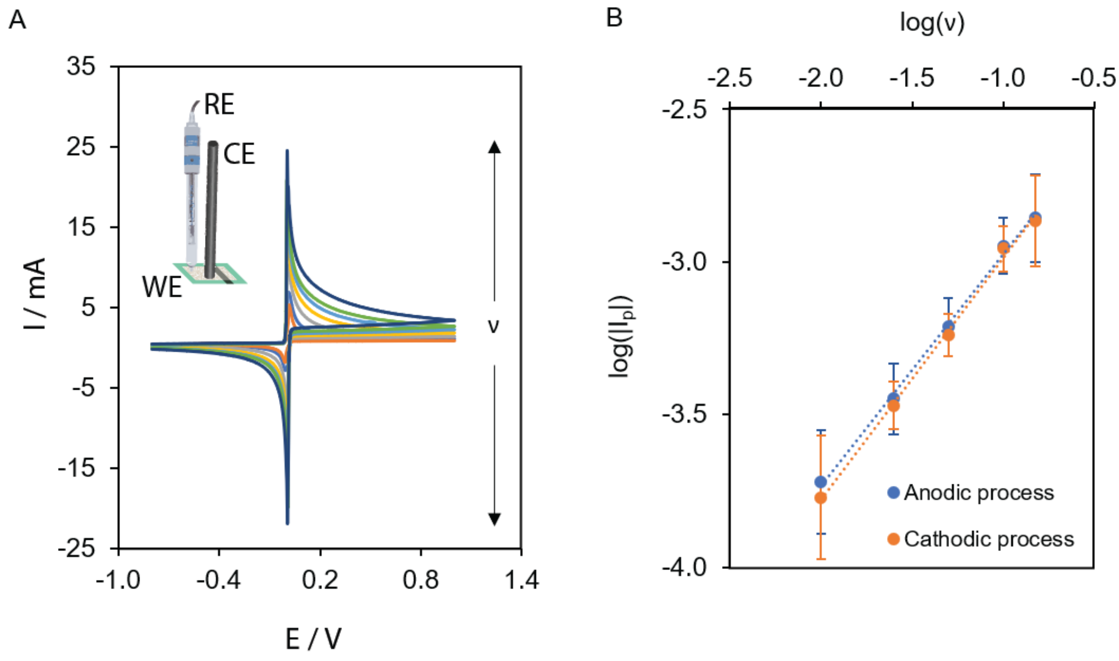

Figure S8: Dependence of the peak current on the scan rate was evaluated by cyclic voltammetry sweeping the potential from –0.8 to +1.0 V vs Ag/AgCl at 10, 25, 50, 75, 100, 150 and 200 mV s^{-1} **(A)**. The gradient of the logarithmic plot peak current intensity vs the scan rate is 0.67 ± 0.08 (R^2 = 0.997) and 0.67 ± 0.09 (R^2 = 0.996), for anodic and cathodic processes respectively (n=5 repeats). The metallized Ag paper working electrode (2 cm x 2 cm of geometric area) has effective areas of 8.2 ± 0.4 cm^2 and 8.6 ± 0.6 cm^2 from the anodic and cathodic data respectively, calculated from the Randles-Sevcik equation **(B)**.

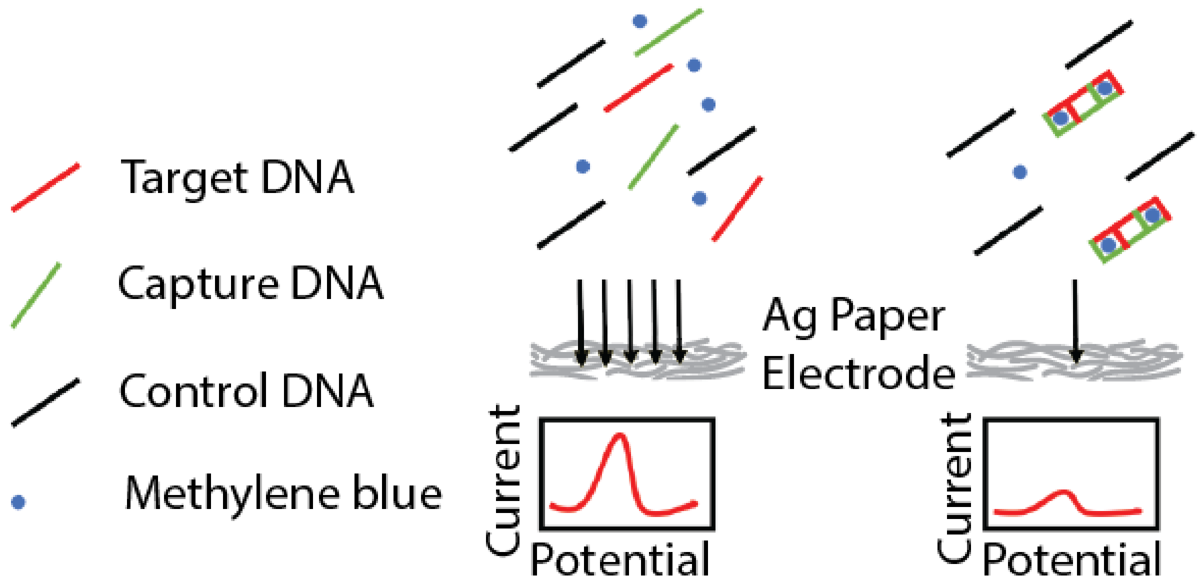

Methylene blue (blue) + H^+ + e^- $\rightleftharpoons$ Leukomethylene blue (colourless) + Cl^-

Figure S9: Schematic illustration of the basis of the electrochemical DNA biosensor. Electrochemical detection is facilitated with methylene blue (MB), a redox-active reporter that binds specifically to the guanine bases during the hybridization of two single strands of DNA. Trapped molecules of MB between the target and capture DNA oligomers reduce the concentration of the free MB in the solution, leading to a decreased redox signal during electroanalysis over SIAM Ag paper electrodes (post-hybridization on **right of figure**). If not trapped by DNA, the high redox signal of the MB to leucomethylene blue reaction would be observed (**left graph**). The equation for this reaction is shown at the bottom of the figure.

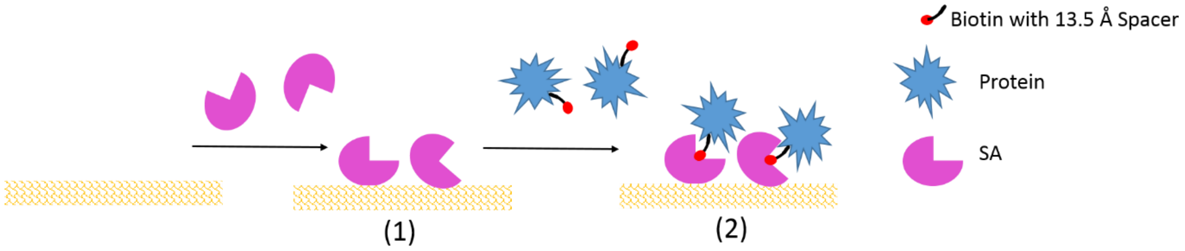

Figure S10: Binding events on metallized Au paper electrode: **(1)** SA was immobilized on Au paper through physical adsorption and **(2)** biotinylated protein was then immobilized on the SA. The enzyme HRP is conjugated through one of its lysine groups to biotin with a short spacer (13.5 Å) and the high affinity ($K_d = 10^{-15}$ M) and fast kinetics of biotin-streptavidin reaction allows capture of biotin-labelled molecules with high specificity.

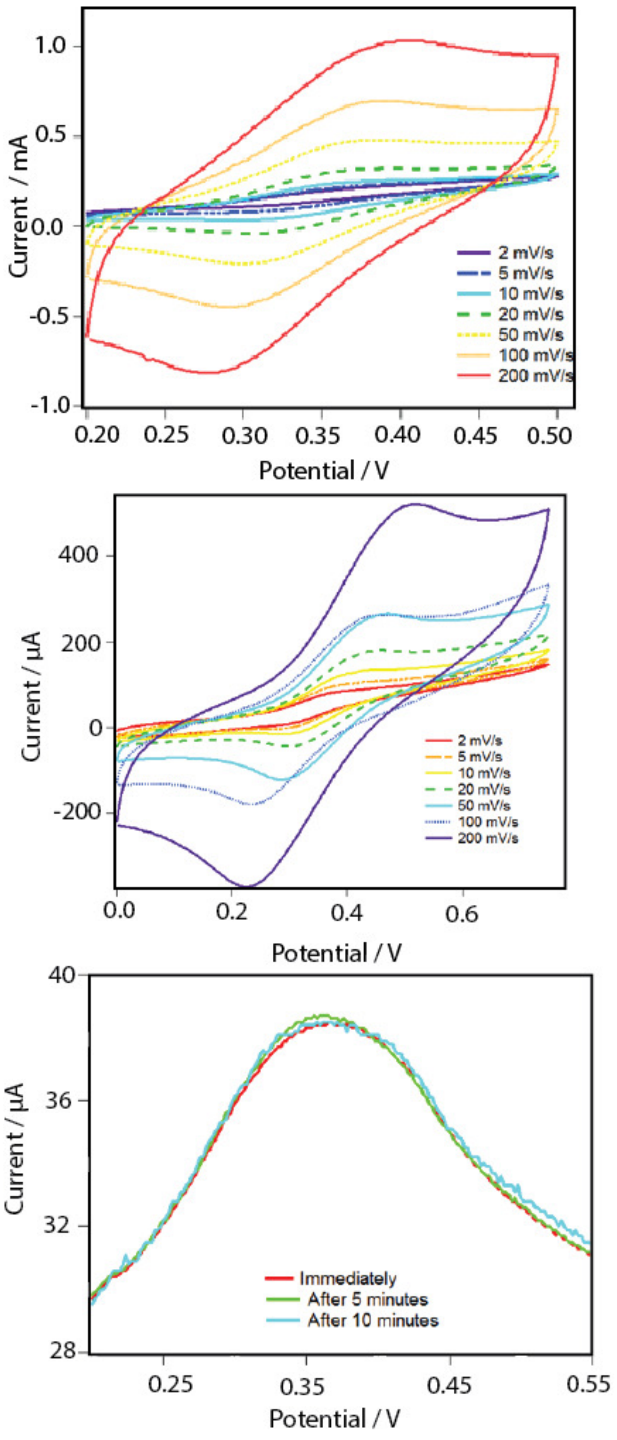

Figure S11: Figure S7 (top) shows CV scans Au paper, not coated with SA, in 2 mM FCA in PBS, at different scan rates ranging from 2 to 200 mV s^{-1}. When SA is immobilized on the Au paper electrode, it blocks access of the FCA redox probe to the electrode surface. This is the same at all scan rates as shown in Figure S7 (middle). Upon addition of biotinylated-HRP, SWV measurements in Figure S7 (bottom) demonstrate the fast kinetics of the biotin-

SA binding, as the measured current signals are stable over a 10 min. period. Concentrations of biotinylated-HRP were determined with Beer's Law using absorbance at 402 nm with an extinction coefficient value $\varepsilon_{402} = 102 \times 10^3\ M^{-1}\ cm^{-1}$.

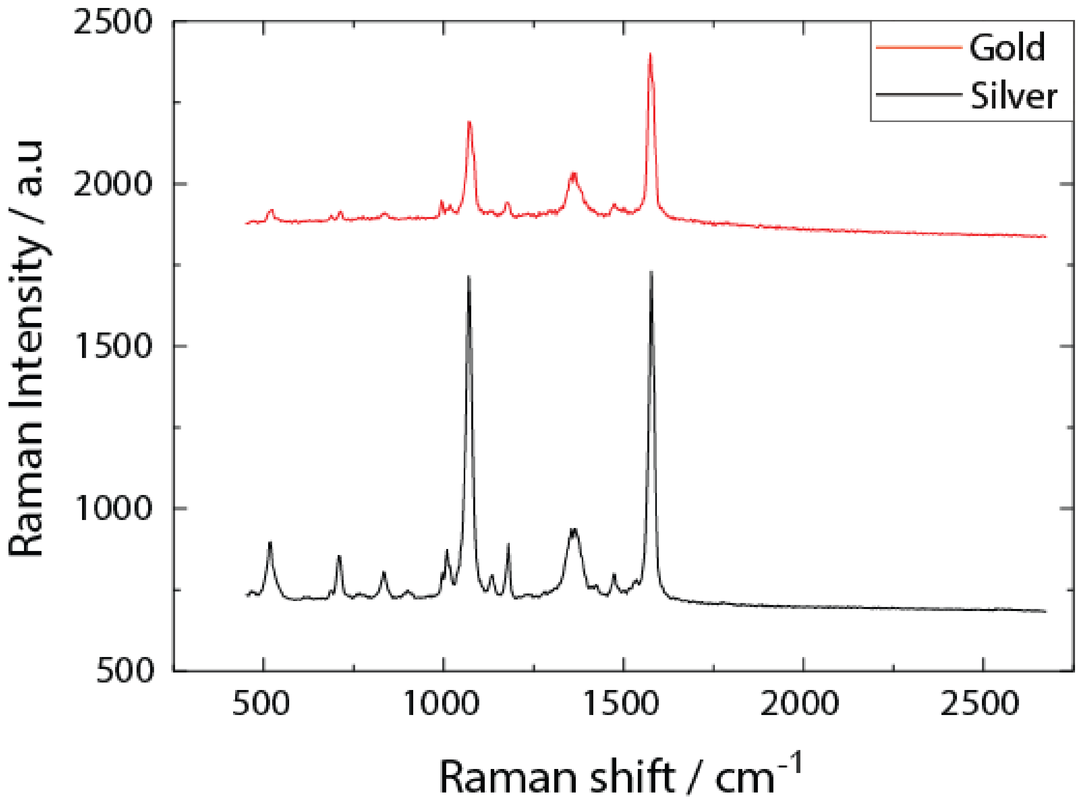

Figure S12: Metallized Ag and Au paper demonstrated as SERS substrates by functionalizing with the Raman marker 4-MBA, characterized by peaks at 1,100 and 1,590 cm^{-1}.

Cost of SIAM: The amount of Ag metal deposited by SIAM of paper is 0.004 g cm^{-2}. This was calculated by removing Ag from SIAM in a HNO_3 bath, and then weighing the difference. The amount of Ag deposited from commercial ink is 0.053 g cm^{-2} (Ag/AgCl 50:50 Paste from Gwent Group, UK). The SIAM process requires, therefore, an order of magnitude less Ag per unit area.

Using SIAM, a square centimeter of paper may be metallized with 0.1 ml 1M $AgNO_3$ salt, costing \$0.40 g^{-1}, therefore, the cost of SIAM of paper is \$0.007 cm^{-2}. With commercial ink costing \$2 g^{-1}, the cost of metallization of paper is \$0.1 cm^{-2}, which is more than an order of magnitude greater.

References

[1] T. R. L. C. Paixao, D. Lowinsohn, M. Bertotti, J. Agric. Food Chem. **2006**, 54, 3072.

[2] M. Lukaszewski, M. Soszko, A. Czerwiński, Int. J. Electrochem. Sci. **2016**, 11, 4442.

[3] S. Trasatti, O. A. Petrii, J. Electroanal. Chem. **1992**, 327, 353.

[4] A. J. Bard, L. R. Faulkner, Electrochemical Methods: Fundamentals and Applications, **2001**.

[5] M. J. Sims, N. V. Rees, E. J. F. Dickinson, R. G. Compton, Sensors Actuators, B Chem. **2010**, 144, 153.
